# Large Immature Intracranial Teratoma in an Infant: A Case Report

**DOI:** 10.7759/cureus.51891

**Published:** 2024-01-08

**Authors:** AJF Da Silva, Carolina Martins Lessa Barreto, Laís Lopes Melo Kummer, Beatriz Profírio Barros Correia, Rosilene Alves Teixeira Ewbank Udihara

**Affiliations:** 1 Pediatric Neurosurgery Division, Santa Mônica Maternity School, Alagoas State University of Health Sciences, Maceió, BRA; 2 Pediatric Neurosurgery Division, General State Hospital (GSH), Maceió, BRA; 3 Teaching Department, Centro Universitário Cesmac (CESMAC), Maceio, BRA; 4 Pathology Department, Laboratório de Anatomia Patológica, Maceio, BRA

**Keywords:** tumours, somatic malignancy, nervous central system, acute hydrocephalus, immature teratoma

## Abstract

Intracranial immature teratomas are rare, highly malignant, and fast-growing with a poor prognosis. We report the case of an infant with a large immature teratoma in the intracranial compartment. A two-month-old child presented to the emergency room with drowsiness and seizures. CT and cranial MRI showed hydrocephalus with a large expansive process in the right cerebral hemisphere extending to the infratentorial compartment, compressing the cerebellum and brainstem. It was then decided to partially resect the lesion. Postoperatively, due to the aggressiveness of the residual tumor, the patient developed complications (status epilepticus, hyperthermia, and electrolyte disorders) and died. Histopathological and immunohistochemical studies confirmed an immature teratoma. Teratomas are a subtype of germ cell tumors. Immature teratomas contain a population of cells that retain embryonic characteristics and tissues with more primitive components derived from all or some of the three germ layers (ectoderm, mesoderm, and endoderm). The prognosis of immature teratomas is associated with the degree of tumor differentiation, and those composed of undifferentiated embryonic tissues have a poor prognosis. This case report illustrates the rare and severe occurrence of a bulky immature cerebral teratoma in an infant. Unfortunately, despite undergoing a planned partial resection, the infant ended up having complications and died. Therefore, due to the size of the lesion in an infant, these cases are always complex when deciding on a surgical approach.

## Introduction

Congenital intracranial tumors are rare and account for only 0.5-1.5% of all infant brain tumors. Teratomas are the tumors with the highest incidence rate at birth [1], with a frequency of 36.4% of all tumors of the central nervous system [2]. Teratomas are composed of multiple tissues and often exhibit asynchronous maturation. They are usually located in the midline and, therefore, are believed to originate in the pineal gland, involving the third ventricle and most often resulting in hydrocephalus [3]. Immature teratomas are rarer (representing 1% of all teratomas), and have a high degree of malignancy, rapid growth, and poor prognosis [4,5].

We report a rare case of an infant with a large immature teratoma extending into multiple intracranial compartments and the challenge of surgical treatment.

## Case presentation

An infant born vaginally from a nonconsanguineous marriage, had an Apgar score of 7 and 8 at the first and fifth minutes, respectively. The mother had had irregular prenatal care. At two months, the infant was admitted to the emergency room due to drowsiness and seizures. On physical examination, the patient was hypoactive and hyporeactive and exhibited macrocephaly (44 cm), namely a wide, bulging, and tense anterior fontanelle. Transfontanellar ultrasound (TFUS) showed significant ventricular dilatation and a heterogeneous area in the right parietal region. CT of the head showed hydrocephalus with a large expansive process in the right cerebral hemisphere extending to the infratentorial compartment and compressing the cerebellum and brainstem. It was decided to treat hydrocephalus immediately by placing a ventriculoperitoneal shunt (VPS).

**Figure 1:**
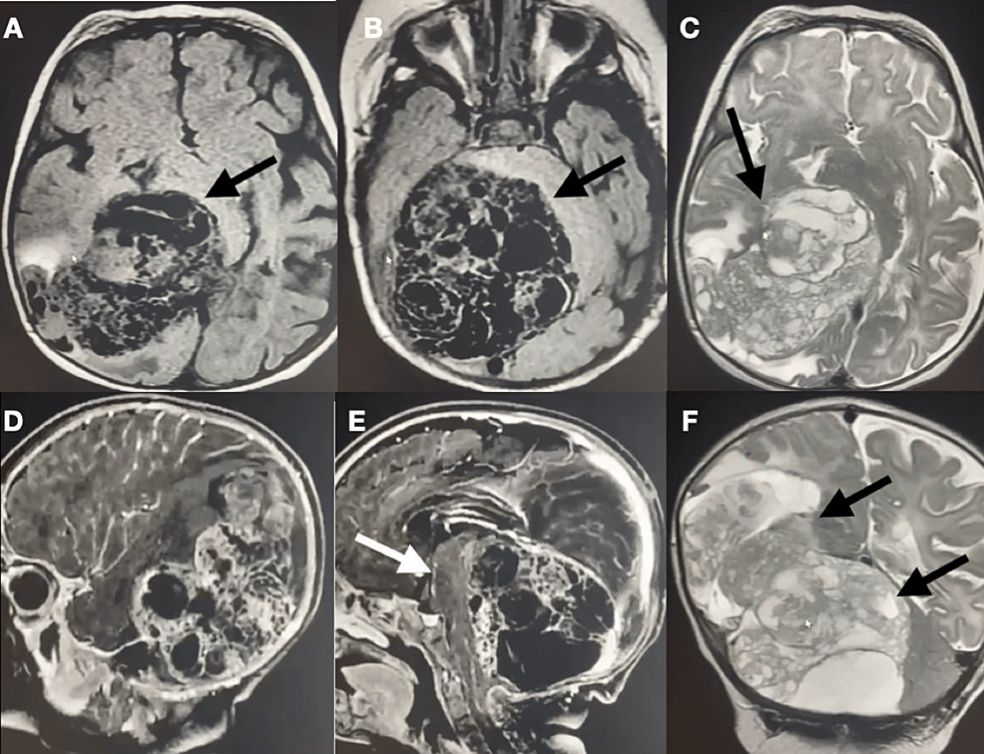
Magnetic Resonance Imaging (A and B) Axial T1, large heterogeneous lesion with significant mass effect with brain stem compression (black arrow); (C) Axial T2, massive infiltrative lesion (black arrow); (D and E) Sagittal view with contrast, tumor compressing the brainstem(white arrow); (F) Coronal T2, tumor in the supra and infratentorial compartment (black arrows)

Subsequently, the infant improved, becoming more awake and active. Then, to better evaluate the lesion, an MRI of the head was performed, which showed a large and very heterogeneous lesion with cystic and solid components (Figure 1A-1B), with some infiltration sites in the posterior region of the right cerebral hemisphere (Figure 1C), extending to the infratentorial compartment (Figure 1F), causing compression in the cerebellum and brainstem (Figure 1D-1E).

**Figure 2:**
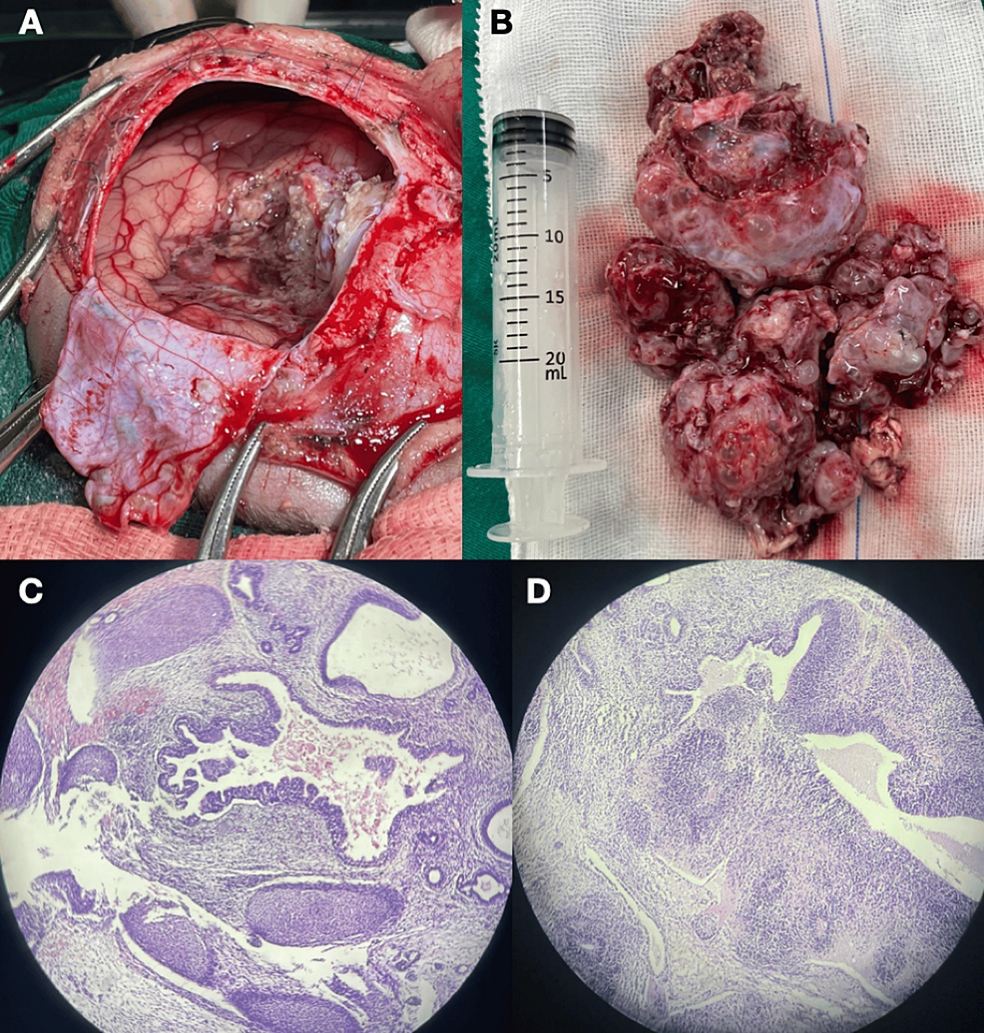
Surgery and histopathological study (A) Craniotomy with exposure of the brain; (B) Part of the lesion resected; (C and D) Histopathology with mature and immature components (H&E, 100x)

Unfortunately, molecular markers had not been made. Therefore, there could be various germ cell tumors and even dermoid tumors as differential diagnoses. Due to the size of the lesion and because the patient was an infant, a right parieto-occipital osteoplastic craniotomy (Figure 2A) was performed with partial resection of the supratentorial part of the lesion (Figure 2B). For the surgical procedure, some criteria were considered: (i) Surgical time, on average three hours, (ii) Blood reserve, even with partial resection, (iii) Control of the infant’s body temperature, and (iv) Experienced anesthesiologist. The infant had a good postoperative evolution and was extubated and off mechanical ventilation on the third day. On the 10th day after surgery, the infant presented with convulsions (which did not respond to diazepam and midazolam), developed status epilepticus, was intubated and sedated with midazolam and thiopental, and 24 hours later had a cardiorespiratory arrest without response to resuscitation maneuvers, and subsequently died. The cause of death could be justified by the aggressiveness of the residual tumor with compression in structures such as the hypothalamus and brain stem, causing, in addition to status epilepticus, hyperthermia, and fluid and electrolyte disorders. The histopathological study showed immature teratoma (Figure 2C-2D) confirmed by immunohistochemistry.

## Discussion

Teratomas are a subtype of germ cell tumors. Approximately 90% of these tumors contain the three layers of germ cells (ectoderm, mesoderm, and endoderm) [1,6]. Intracranial teratomas are classified according to the histological variant: (i) mature teratoma containing well-differentiated tissue with the three germ layers, (ii) immature teratoma containing a population of cells that retain embryonic characteristics and tissues with more primitive components derived from all or some of the three germ layers, and (iii) teratoma with somatic malignancy is the malignant transformation of a teratomatous component (mature or immature) into a non-germ cell malignancy (rhabdomyosarcoma type) [3,7,8]. In the reported case, the histological study showed elements of the three germ cell layers (ectoderm, mesoderm, and endoderm) represented by mature elements (cartilage, squamous epithelium, and mucinous glandular epithelium) and immature elements (primitive neuroepithelium), suggesting a diagnosis of immature teratoma, which was confirmed by immunohistochemistry.

Teratomas are midline tumors that affect the pineal region, suprasellar region, quadrigeminal plate, third ventricle wall, and cerebellar vermis. They can be found in locations outside the midline, namely the basal ganglia, cerebellopontine angle, and cavernous sinus [8]. The differential diagnosis of midline intracranial neoplasms includes germ cell tumors (germinoma, choriocarcinoma, embryonal carcinoma, and endodermal sinus tumor) and dermoid tumors [9]. In the case described here, it is unclear whether it was a tumor with an epicenter in the hemisphere extending to midline structures or a tumor with an epicenter in the midline (most likely) extending to the cerebral hemisphere.

Teratomas may be associated with macrocephaly, hydrocephalus, and polyhydramnios. Patients have seizures in approximately 20% of cases. Often the pressure of the tumor on important structures such as the brainstem causes ischemia or neuronal changes even before the development of hydrocephalus [6,10]. In the present case, the infant presented with macrocephaly, seizures, and signs of intracranial hypertension.

The diagnosis can be made prenatally from the 20th week of gestation through an ultrasound (US) showing obstructive hydrocephalus caused by the tumor. CT and MRI provide more detail than US, especially for differential diagnosis. CT shows calcifications while MRI allows to better delineate the extent of the lesion, especially in the posterior fossa [8,10]. With regard to molecular marker analysis, elevated serum alpha-fetoprotein (AFP) is associated with more immature teratomas [4]. Little is known about the genetic and molecular etiopathogenesis of immature teratomas. It is known that DNA methylation levels in mature and immature teratomas are similar, but there are some aberrant methylation levels in immature teratomas [11]. In the present case, TFUS was initially performed, followed by a head CT, and then a head MRI was performed to further evaluate the condition. Tumor markers were not requested.

The approach to teratoma treatment is maximal resection and adjuvant chemotherapy (the most common regimen consists of cis‑platinum, Bleocin, and etoposide or cyclophosphamide, combined with taxol) and radiotherapy, especially for immature teratomas, which can be curative in benign and small teratomas. The prognosis of immature teratoma is associated with the degree of tumor differentiation, i.e., those composed of undifferentiated embryonic tissues have a worse prognosis [12,13]. In immature teratomas, compared with mixed germ cell tumors, fewer genetic alterations were found [11]. In the case reported here, the tumor was shown to be aggressive, with a large mass effect, and therefore difficult to manage in a two-month-old infant. Perhaps cellular and molecular biology or genetic engineering will shed more light on the behavior of this tumor. This could avoid the diagnosis of an extensive tumor lesion at an advanced stage.

## Conclusions

Teratomas are germ cell tumors with a high incidence rate at birth. This case report describes the rare and serious occurrence of a large immature cerebral teratoma in an infant. All difficulties in the surgical management of the case were discussed. Unfortunately, even with a planned partial resection, the patient ended up experiencing complications and died. Therefore, due to the size of the lesion in an infant, these cases are always complex when deciding on a surgical approach.
